# Mimicking chronic glaucoma over 6 months with a single intracameral injection of dexamethasone/fibronectin-loaded PLGA microspheres

**DOI:** 10.1080/10717544.2022.2096712

**Published:** 2022-07-29

**Authors:** Alba Aragón-Navas, María J. Rodrigo, David Garcia-Herranz, Teresa Martinez, Manuel Subias, Silvia Mendez, Jesús Ruberte, Judit Pampalona, Irene Bravo-Osuna, Julian Garcia-Feijoo, Luis E. Pablo, Elena Garcia-Martin, Rocío Herrero-Vanrell

**Affiliations:** a Complutense University, Innovation, Therapy and Pharmaceutical Development in Ophthalmology (InnOftal) Research Group, UCM 920415, Department of Pharmaceutics and Food Technology, Faculty of Pharmacy, Complutense University of Madrid Spain, Health Research Institute of the San Carlos Clinical Hospital (IdISSC), Madrid, Spain; b Department of Pharmaceutics and Food Technology, Faculty of Pharmacy, Complutense University of Madrid, Madrid, Spain; c Research Institute of the San Carlos Clinical Hospital (IdISSC), Grupo de Investigación Innovación Farmacéutica en Oftalmología, Madrid, Spain; d Instituto de Investigación Sanitaria de Aragón, Hospital Universitario Miguel Servet, Universidad de Zaragoza, Zaragoza, Spain; e National Ocular Pathology Network (OFTARED) Carlos III Health Institute, Madrid, Spain; f Center of Animal Biotechnology and Gene Therapy (CBATEG), Universitat Autònoma de Barcelona, Bellaterra, Spain; g CIBER de Diabetes y Enfermedades Metabólicas Asociadas (CIBERDEM), Madrid, Spain; h Department of Animal Health and Anatomy, School of Veterinary Medicine, Universitat Autònoma de Barcelona, Bellaterra, Spain;; i Department of Ophthalmology, San Carlos Clinical Hospital, Health Research Institute of the San Carlos Clinical Hospital (IdISSC), Madrid, Spain

**Keywords:** Glaucoma animal model, PLGA microspheres, multiloaded, intraocular pressure, neurodegeneration

## Abstract

To create a chronic glaucoma animal model by a single intracameral injection of biodegradable poly lactic-co-glycolic acid (PLGA) microspheres (Ms) co-loaded with dexamethasone and fibronectin (MsDexaFibro).

MsDexaFibro were prepared by a water-in-oil-in-water emulsion method including dexamethasone in the organic phase and fibronectin in the inner aqueous phase. To create the chronic glaucoma model, an interventionist and longitudinal animal study was performed using forty-five Long Evans rats (4-week-old). Rats received a single intracameral injection of MsDexafibro suspension (10%w/v) in the right eye. Ophthalmological parameters such as clinical signs, intraocular pressure (IOP), neuro-retinal functionality by electroretinography (ERG), retinal structural analysis by optical coherence tomography (OCT), and histology were evaluated up to six months. According to the results obtained, the model proposed was able to induce IOP increasing in both eyes over the study, higher in the injected eyes up to 6 weeks (p < 0.05), while preserving the ocular surface. OCT quantified progressive neuro-retinal degeneration (mainly in the retinal nerve fiber layer) in both eyes but higher in the injected eye. Ganglion cell functionality decreased in injected eyes, thus smaller amplitudes in PhNR were detected by ERG.

In conclusion, a new chronic glaucoma animal model was created by a single injection of MsDexaFibro very similar to open-angle glaucoma occurring in humans. This model would impact in different fields such as ophthalmology, allowing long period of study of this pathology; pharmacology, evaluating the neuroprotective activity of active compounds; and pharmaceutical technology, allowing the correct evaluation of the efficacy of long-term sustained ocular drug delivery systems.

## Introduction

Glaucoma is a multifactorial neurodegenerative disorder, leading cause of blindness with an estimated 60 million people worldwide currently suffering and it will be around 118 millions in 2040 (Quigley & Broman, 2006; Tham et al., 2014). It is produced by the gradual death of retinal ganglion cells (RGC) which leads to irreversible vision loss, although recent studies have also suggested the involvement of other retinal cells as photoreceptors or activated glia (Asaoka et al., 2017; Ramirez et al., 2017; Ashimatey et al., 2018). Intraocular pressure (IOP) is the major modifiable risk factor strongly associated with the onset and progression of the disease (Gaasterland et al., 2000). For this reason, the usual treatment of glaucoma consists in the frequent instillation of hypotensive drugs (Conlon et al., 2017); however, it is important to remark that the return to normal intraocular pressure values does not always stop the retinal degeneration (Pascale et al., 2012) and that, in addition, in many other cases these same failures in the retina and optic nerve occur in normotensive patients (Anderson, 2003; Lestak et al., 2018). Sadly, there is not yet any optimal treatment for this and any other retinal neurodegenerative diseases.

Degenerative diseases of the retina begin with primary damage, for example of the axons of retinal ganglion cells in the case of glaucoma. When the damaged cell dies, it releases neurotoxic compounds in the vicinity that damage the adjacent neurons that also end up dying in what is known as “secondary neurodegeneration” (Ritch, 2000). Several decades ago, some researchers postulated the idea of “neuroprotection” as a complementary treatment in degenerative diseases (Weinreb & Levin, 1999). At the level of the retina neuroprotection can be defined as: “Prevention or slowing down of the loss of functional integrity of the cells of the retina, their axons and their axonal connections to maintain and stabilize the vision of patients as much as possible” (Ritch, 2000). Pardue et al. describe this neurodegeneration of the retina in 3 stages: an adaptive first step where there is only oxidative stress and neuronal dysfunction, a second stage, the “Early pathology”, where retinal structures begin to damage but still reversibly, and a third stage of "Late pathology" where blindness appears and where the damage is already irreversible. According to those authors, “starting neuroprotective treatments at the first signs of the retinal disease would provide the most benefit in preserving vision” (Pardue & Allen, 2018).

In recent decades, different neuroprotective pharmacological strategies have been explored (Thanos & Emerich, 2005; Masuda et al., 2017; Avotri et al., 2019; Fernández-Albarral et al., 2019; Stankowska et al., 2019; Naik et al., 2020). However, one of the main problems to evaluate the efficacy of neuroprotective treatments resides in the absence of a chronic and slow degeneration glaucoma animal model that simulates the conditions found in the human glaucomatous eyes (Pang & Clark, 2020). In general, it is very difficult to obtain optimal animal models of retinal pathologies. For example, in the case of glaucoma, there are the so-called “acute models”, in which a fast retinal degeneration occurs, either due to genetic failures (Schlamp et al., 2006), to the direct intravitreal injection of toxic compounds (Kitaoka et al., 2006), or to the direct damage of the optic nerve for example by crushing it (Sharma et al., 2014). Despite their utility for neuroprotective studies, these acute models: (1) Do not reproduce the reality of retinal chronic pathologies in human beings; (2) Do not allow testing the long term efficacy of new therapies because the retina is almost directly in the last step of the diseases from the very beginning of the study; and (3) It is difficult to assess the potential of modified release systems so in a few days or weeks the retina is completely ruined (Cuenca et al., 2014; Smedowski et al., 2014; Pang & Clark, 2020). There are also the so-called "chronic models”, which present a more progressive retinal degradation based on the increase in IOP secondary to a limitation of aqueous humor outflow. In rodent models, the reduction in aqueous humor drainage is typically obtained, either through cauterization, ligature and/or sclerosis of episcleral veins (Morrison et al., 1997; Morgan & Tribble, 2015; Dey et al., 2018) either by mechanical blockage of the trabecular meshwork (TM) with non-biodegradable particles injected in the anterior chamber (Urcola et al., 2006), through the use of corticoids which reduce aqueous humor outflow when inhibiting cellular phagocytosis thus avoiding cleaning the waste channels in the TM (Clark & Wordinger, 2009; Patel et al., 2017), or by fibronectin expression in a genetically modified mouse model altering the extracellular matrix of the TM (Roberts et al., 2020). All the previous need repeated interventions on the animal, damage aggressively or even modify their nature.

Thus far, no research group has achieved the creation of an animal model which simulates open-angle glaucoma combining the last three different strategies. With this idea in mind, we recently proposed an initial animal model of glaucoma by using intracameral injections of non-loaded PLGA microspheres. The weekly injection of blank microspheres produced a sustained and prolonged ocular hypertension (OHT) over six months due to a progressive physical blockage of the trabecular meshwork. This maintained OHT produced a very progressive degeneration of the retina (Garcia-Herranz et al., 2021; Rodrigo et al., 2021). In a second study, dexamethasone loaded PLGA microspheres also generated OHT and neuroretinal degeneration by only two times injections (Rodrigo et al., 2021). After those initial approaches, in the present work, we propose a new model of chronic glaucoma produced by intracameral injection of dexamethasone/fibronectin co-loaded PLGA microspheres. This study aims to establish whether lower quantities of sustained and simultaneous co-release of those two active compounds from PLGA microspheres in the anterior segment of the eye, combined with the mechanical blockage produced by PLGA microspheres themselves, would promote a progressive OHT and subsequent retinal chronic degradation, only using a single injection. The follow up of animals in terms of retinal structure and function is performed for six months.

## Materials

Dexamethasone (DX) was supplied by Sigma-Aldrich (St. Louis Mo., USA) (purity >98%). Poly(D,L-lactide-co-glycolide) (PLGA) 50:50 (Inherent viscosity: 0.16–0.24 dL/g) was purchased from Evonik Industries (Essen, Germany). Polyvinyl alcohol 67,000 g/mol (PVA) was obtained from Merck KGaA (Darmstadt, Germany) and methylene chloride from PanReac AppliChem (Barcelona, Spain). Fibronectin (FN), fibronectin ELISA and reactants (Reagent Diluent DY995, Wash Buffer WA126, Stop Solution DY994 and Substrate Reagent Pack DY999) were purchased from R&D Systems (Minneapolis, MN, USA).

### Manufacture of dexamethasone/fibronectin-loaded PLGA microspheres

**Figure 1.**
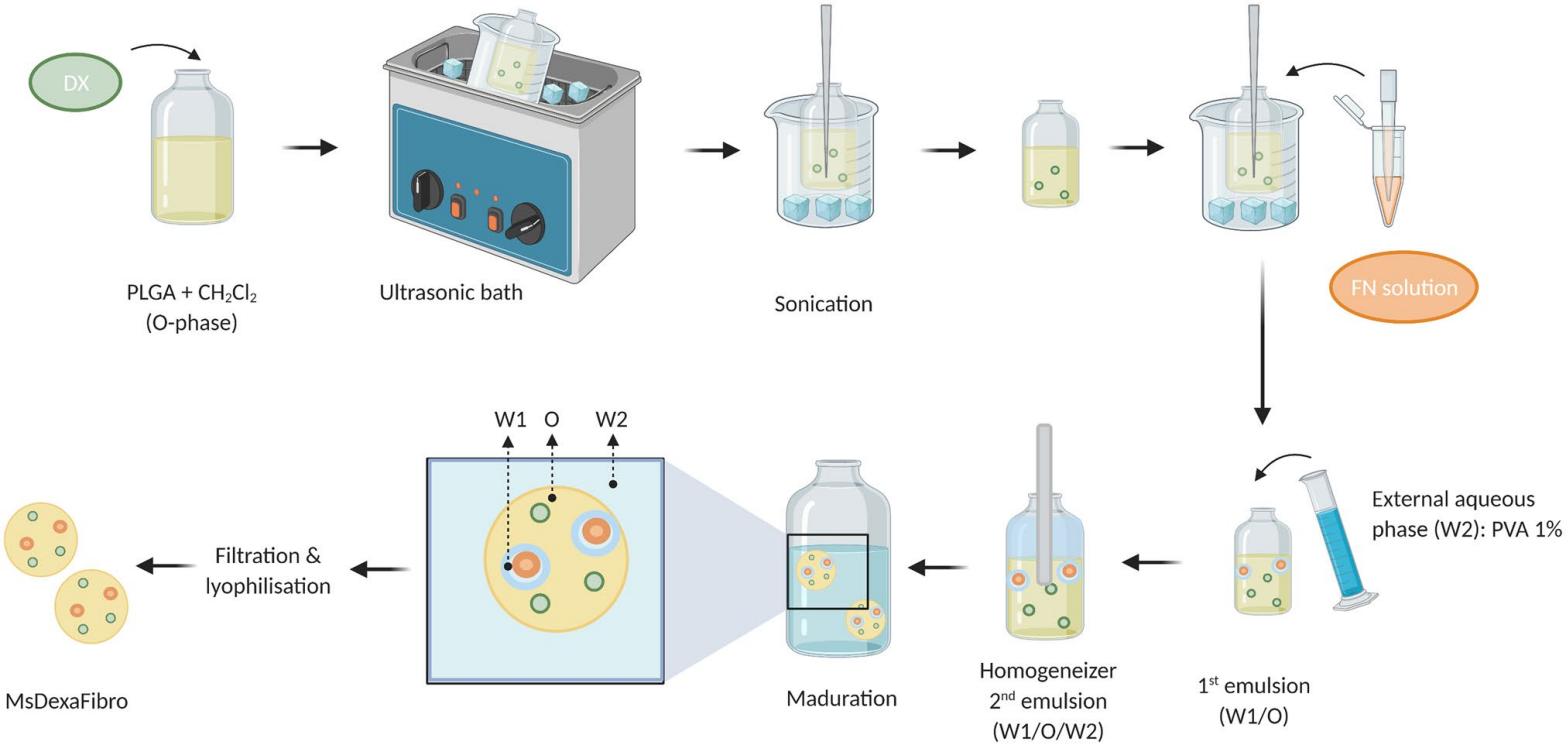
Elaboration scheme of dexamethasone-fibronectin co-loaded PLGA microspheres. Created with BioRender.com.

The water-in-oil-in-water (W/O/W) double emulsion solvent followed by extraction-evaporation technique was employed for dexamethasone/fibronectin-loaded PLGA microspheres elaboration. In brief, micronized DX (40 mg) was added to a polymeric organic solution (400 mg of PLGA dissolved in 2 mL of methylene chloride). The suspension was homogenized by ultrasonication in an ice-water bath (Ultrasons; J.P. Selecta, Barcelona, Spain) for 5 minutes and sonication (Sonicator XL; Heat Systems, Inc., Farmingdale, NY, USA) for 1 additional minute in an ice-water bath. The inner aqueous phase composed of 20 µL of FN aqueous solution (containing 42.8 µg of fibronectin) was added to this organic phase and emulsified by sonication (Sonicator XL; Heat Systems, Inc., Farmingdale, NY, USA) for 30 seconds at 4 °C to create the initial W_1_/O emulsion. Afterwards, 5 mL of PVA solution 1% (w/v) were added to the mentioned W_1_/O emulsion and the mixture was emulsified (Polytron®RECO, Kinematica, GmbHT PT3000, Lucerna, Switzerland) at 7,000 rpm for 1 minute to form the final W_1_/O/W_2_ emulsion that was finally added to 100 mL of PVA solution 0.1% (w/v) to get droplets hardening by organic solvent evaporation under stirring at room temperature for 3 hours. After that, Ms were washed with MilliQ® water to remove surface PVA. The desired granulometric size fraction (20–10 µm) was collected by sieving, freeze-dried (Freezing: −60 °C/15 min, drying: −60 °C/12h/0.1 mBar) and storage at −30 °C in dry conditions (Figure 1).

### Dexamethasone/fibronectin-loaded PLGA microspheres characterization

#### Production yield percentage (PY %)

The production yield percentage of the chosen granulometric fraction was calculated according to the following Eq. (1):

(1) $$PY\%=\frac{\mathrm{WeightofMs}(W_{1})}{\mathrm{weightofpolymer}(W_{2})+\mathrm{weightofDX}(W_{3})+\mathrm{weightofFN}(W_{4})}x100\;$$

#### Morphological evaluation

Scanning electron microscopy (SEM, Jeol, JSM-6335F, Tokyo, Japan) was employed for the observation of the external surface of dexamethasone/fibronectin-loaded PLGA microspheres after gold sputter-coating.

Transmission electron microscopy (TEM, Jeol 1010, Tokyo, Japan) was used for the assessment of the internal structure. Ms were embedded in a synthetic resin medium (Spurr Low Viscosity Embedding Kit) and cut in slides (50–70 nm) by Reichert Ultracut S ultramicrotome (Leica Microsystems Inc, Wetzlar, Germany).

#### Mean particle size and particle size distribution

Dual light scattering (Microtrac® S3500 Series Particle Size Analyzer, Montgomeryville, PA, USA) was the technique employed to analyze particle size and particle size distribution. The volume mean diameters (± standard deviation) obtained from 3 measurements were calculated and expressed as mean particle size.

#### Dexamethasone quantification by HPLC/MS

Dexamethasone was quantified using a HPLC/MS system consisting of a liquid chromatography instrument (Waters 1525 binary HPLC pump and Waters 2707 autosampler) connected to a MS detector (Waters 3100 single quadrupole mass spectrometer). A guard column (4 µm, 3.9 mm x 20 mm) and a Nova-Pak C18 column (4 µm, ID 2.1 mm x 150 mm) were used in-line at 45 °C in the HPLC instrument. The MS detector was connected to the system via Empower 2 (Waters, Milford, USA). For DX detection, the positive ion mode was chosen for the ESI source. Selected ion recording (SIR) DX mass (m/z) 393.40 was obtained under mass spectrometer source conditions of 3.5 kV electrospray voltage. The conditions for nebulization were 150 L/h flow rate, 120 °C source temperature, 3 V extractor voltage and for desolvation were 500 L/h flow rate, 350 °C desolvation temperature. Nitrogen gas (> 99.999%) was employed in nebulization and desolvation. For encapsulation efficiency and release from Ms, an isocratic method was developed which was composed of 50% ammonium acetate 15 mM/1 mL formic acid in MilliQ® water and 50% acetonitrile (flow rate: 0.3 mL/min).

### Fibronectin quantification by enzyme-linked immunosorbent assay (ELISA)

Fibronectin quantification was performed by ELISA technique using a Fibronectin ELISA Kit (DuoSet® Human Fibronectin DY1918-05, R&D Systems, Minneapolis, MN, USA).

#### Dexamethasone encapsulation efficiency from dexamethasone/fibronectin-loaded PLGA microspheres

1 mg of dexamethasone/fibronectin-loaded PLGA Ms was dissolved in 2.5 mL of methylene chloride. Afterwards, 6 mL of methanol (MeOH) was added to cause the polymer precipitation, remaining the drug dissolved in the methylene chloride:methanol mixture. This organic solution was isolated from the solid polymer by centrifugation (5,000 rpm, 5 minutes, 20 °C). The supernatant was collected, filtered (0.22 µm) and analyzed by the HPLC/MS method previously described.

#### Dexamethasone and fibronectin *in vitro* release studies from dexamethasone/fibronectin-loaded PLGA microspheres

2.5 mg of dexamethasone/fibronectin-loaded PLGA Ms were incubated in 1 mL of release media composed by a phosphate-buffered saline (PBS, pH 7.4) solution including sodium azide (0.02% (w/v) (*n* = 4). The so-prepared samples were located in a water shaker bath (100 rpm, 37 °C, Memmert Shaking Bath, Memmert, Schwabach, Germany). At pre-set times samples were gently centrifugated (5,000 rpm for 5 min, 20 °C). The supernatant was extracted, filtered (0.22 μm) and used for dexamethasone quantification by HPLC/MS employing the method previously mentioned. The formed pellet was resuspended in the same volume of fresh release media to continue the study until the following sampling time-point.

In parallel, the same amount of dexamethasone/fibronectin-loaded PLGA Ms was suspended in phosphate-buffered saline (1 ml, PBS, pH 7.4) including sodium azide (0.02% (w/v) and bovine serum albumin (BSA) (1% (w/v)). The suspension was placed in a water shaker bath (100 rpm, 37 °C, Memmert Shaking Bath, Memmert Schwabach, Germany) (*n* = 2). Similarly, at pre-set times, the so-prepared samples were centrifuged (5,000 rpm, 5 minutes, 20 °C), the supernatants removed for FN quantification by ELISA and the remaining microspheres refilled with fresh PBS/azide/BSA media.

#### Animal’s welfare and anesthesia

The work with animals was carried out in the experimental surgery service department of the Biomedical Research Center of Aragon (CIBA). The experiment was previously approved by the Ethics Committee for Animal Research (PI34/17) and carried out in strict accordance with the ARVO Statement for the Use of Animals in Ophthalmic and Vision Research.

The study was carried out in 45 (40% males, 60% females) Long Evans rats with 4-week-old of age and weights ranged from 50 to 100 grams at the beginning of the study. Animals were housed in standard cages with water and food *ad libitum*, in 12-hours dark-light cycled rooms with temperature (22 °C) and relative humidity (55%) controlled conditions. IOP measurements were recorded under gas anesthesia with a mixture of 3% sevoflurane gas and 1.5% oxygen and the same gas anesthesia with subcutaneous analgesia (dilution 1/10 of buprenorphine (0.05 mg/kg)) was used for performing OHT injections. General anesthesia by intraperitoneal injections (60 mg/Kg of Ketamine + 0.25 mg/Kg of Dexmedetomidine) was used for electroretinogram (ERG) and optical coherence tomography (OCT) and mydriatic eye drops with tropicamide 10 mg/ml and phenylephrine 100 mg/ml, (Alcon Cusí® SA, Barcelona, Spain) to fully dilate. Surgical and sedative procedures were realized under temperature control with warm pads, antisepsia conditions, topical anesthesia with tetracaine 1 mg/ml + oxibuprocaine 4 mg/ml (Anestesico doble Colircusi®, Alcon Cusí® SA, Barcelona, Spain) and antibiotic with ofloxacin 3 mg/ml (Exocin Colircusi®, Alcon Cusí® SA, Barcelona, Spain) eye drops and lubrication on eyes with hypromellose 2% (Methocel® OmniVision, Germany) or erythromycin 5 mg/g (Oftalmolosa Cusí® eritromicina, Alcon Cusí® SA, Barcelona, Spain) and after procedures animals were let recovering in enriched 2.5% oxygen atmosphere.

#### Injection procedure for ocular hypertension induction

Forty-five rats received 2 microliters PLGA MsDexaFibro suspension (10% w/v) into the anterior chamber of the right eye (RE) using a 10-μl-Hamilton® syringe and glass micropipette by a corneal superotemporal puncture at baseline. To avoid reflux it is recommended placing the rat on its left side and covering the eye puncture with a surgical sponge.

#### Clinical signs and intraocular pressure measurements

Ophthalmological clinical signs such as ocular hyperemia, cornea alteration, infection or intraocular inflammation were weekly evaluated; as well as IOP measured with the rebound tonometer Tonolab® (Tonolab; Tiolat Oy Helsinki, Finland). The value of the IOP was the average of three consecutive measurements, which resulted from the average of 6 rebounds. IOP was always recorded in the mornings (9 to 13 am).

#### *In vivo* neuro-retinal examination

##### Electroretinography

Functionality of neuro-retinal structures was studied using electroretinography (ERG) (Roland consult® RETIanimal ERG, Germany) by full-field scotopic ERG and Photopic Negative Response (PhNR) protocols at baseline, 12 and 24 weeks. To perform the scotopic ERG test animals were dark-adapted for 12 hours and pupils fully dilated. Active electrodes were placed on the cornea, references at both sides under the skin and the ground electrode nearby its tail. Electrode impedance was accepted with a difference <2kΩ between electrodes. Both eyes were simultaneously tested by a Ganzfeld Q450 SC sphere with white LEDs flashes for stimuli and seven steps with increasing intensity of luminance and intervals were performed. Scotopic test examined rod response: step 1: −40 dB, 0.0003 cds/m^2^, 0.2 Hz [20 recordings averaged]; step 2: −30 dB, 0.003 cds/m^2^, 0.125 Hz [18 recordings averaged]; step 3: −20 dB, 0.03 cds/m^2^, 8.929 Hz [14 recordings averaged]; step 4: −20 dB, 0.03 cds/m^2^, 0.111 Hz [15 recordings averaged]; step 5: −10 dB, 0.3 cds/m^2^, 0.077 Hz [15 recordings averaged]; mixed rod–cone response: step 6: 0 dB, 3.0 cds/m^2^, 0.067 Hz [12 recordings averaged]; and oscillatory potentials: step 7: 0 dB, 3.0 cds/m^2^, 29.412 Hz [10 recordings averaged]). The PhNR protocol was performed after light adaptation to blue background (470 nm, 25 cds/m^2^), and a red LED flash (625 nm, −10 dB, 0.30 cds/m^2^, 1.199 Hz [20 recordings averaged]) was used as stimuli. Latency (in milliseconds) and amplitude (in microvolts) were studied in a, b and PhNR waves.

##### Optical coherence tomography

Neuro-retinal structures were studied with the Spectralis OCT device (Heidelberg® Engineering, Germany) and with a contact lens adapted on the rat cornea to get higher quality acquisitions. It was performed at baseline and at 2, 4, 6, 8, 12, 18 and 24 weeks after the OHT injection. Protocols such as full Retina thickness posterior pole (R), Retina Nerve Fiber Layer (RNFL) and Ganglion Cells Layer (GCL) with automatic segmentation were used. These protocols analyzed an area of 3 mm around the center of the optic disk by 61 b-scans and subsequent follow-up examinations were acquired at this same location using the eye tracking software and follow-up application. The Retina and GCL were analyzed by mimicking the 9 ETDRS areas which included a central (C) 1 mm circle centered in the optic disk, (though no fovea exists in rats) and inner (inferior -II-, superior -IS-, nasal -IN-, temporal -IT-) and outer (inferior -OI-, superior -OS, nasal -ON-, temporal -OT-) rings measuring 2 and 3 mm in diameter, respectively, as well as total volume (TV). The RNFL protocol provides measurements of the 6 peripapillary sectors (inferotemporal -IT-, inferonasal -IN-, superotemporal -ST-, superonasal -SN-, nasal -N- and temporal -T-). Full retinal thickness posterior pole (R) comprises from the inner limiting membrane to the retinal pigment epithelium; RNFL from the inner limiting membrane to the GCL boundaries; and GCL from RNFL to the inner nuclear layer boundaries.

Biased examinations were rejected or manually corrected if the algorithm had obviously failed.

### Histology

Animals were euthanized under humanity conditions with an intracardiac injection of sodium thiopental (25 mg/ml) under general anesthesia and eyes were immediately enucleated. Paraffin-embedded eyes were sectioned (5 µm) along the eye axis, deparaffinized and rehydrated. After several washes in phosphate-buffered saline (PBS), sections were incubated overnight at 4 °C with mouse anti-Brn3a (Santa Cruz Biotechnology, Heidelberg, Germany) at 1:50 dilution. After washing the sections in PBS, slides were incubated for 2 hours at room temperature with biotinylated horse anti-mouse at 1:50 dilution (Vector Laboratories, Burlingame, CA, USA). Then, incubation with ABC-HRP (Thermo Fisher Scientific, Waltham Massachusetts, USA) at 1:50 dilution at room temperature was performed. Finally, sections were stained with DAB (Sigma-Aldrich) for 3 minutes and counterstained with Harrys Hematoxylin (Sigma-Aldrich) for 20 minutes at room temperature. Ganglion cells were counted in radial sections of the retina, along 2 mm of a linear region of the ganglion cell layer, at four areas, two in both sides of the optic nerve head. Images were analyzed by an operator blinded to treatment groups. Statistical analysis of the number of ganglion cells was conducted in R (v. 3.6.0) using a paired t-test. The results are shown as mean ± SEM. Values of *p* < 0.05 were considered statistically significant. Procedural immunohistochemistry controls were carried out by omitting the primary antibody in a sequential tissue section.

To analyze the anterior segment of the eye and the location of the microspheres, sections were deparaffinized and stained with Hematoxylin/Eosin or only with Harris Hematoxylin and then observed by differential interference contrast (Nomarski) microscopy. In addition, paraffin sections were stained with the fluorescent dye BODIPY (Invitrogen, Carlsbad, CA, USA) at 1:50 dilution and nuclear counterstained using Hoechst (Sigma-Aldrich) at 1:100 were assessed. For fluorescence image acquisition, a laser scanning confocal microscope (TCS SP5; Leica Microsystems GmbH, Heidelberg, Germany) was used. Image analyses were performed with Fiji (Schindelin et al., 2012).

### Statistical analysis

Data were recorded in an Excel database and statistical analysis was performed using SPSS software version 20.0 (SPSS Inc., Chicago, IL). The Kolmogorov-Smirnov test was used to assess sample distribution. Given the parametric distribution of the data, Student’s t-test was used to evaluate the differences between eyes, and a paired Student’s t-test was used to compare the changes recorded in each eye over the study period. All values were expressed as means ± standard deviations. Values of *p* < 0.05 (expressed as *) were considered to indicate statistical significance. The Bonferroni correction for multiple comparisons was also calculated to avoid a high false-positive rate. The level of significance for each variable was established according to Bonferroni calculations (expressed as ^#^).

## Results

### Production yield

Ms showed a production yield of 55.14% for the selected 20–10 µm fraction. The particle size distribution resulted unimodal with a mean particle size value of 14.81 ± 0.30 µm.

### Encapsulation efficiency

The dexamethasone encapsulation efficiency measurements lead to 79.13 ± 2.64% of the initial drug included during the preparation procedure (71.94 ± 2.40 µg DX/mg Ms). Unfortunately, the fibronectin lability made impossible the real quantification of the loaded protein.

### Morphological evaluation

**Figure 2.**
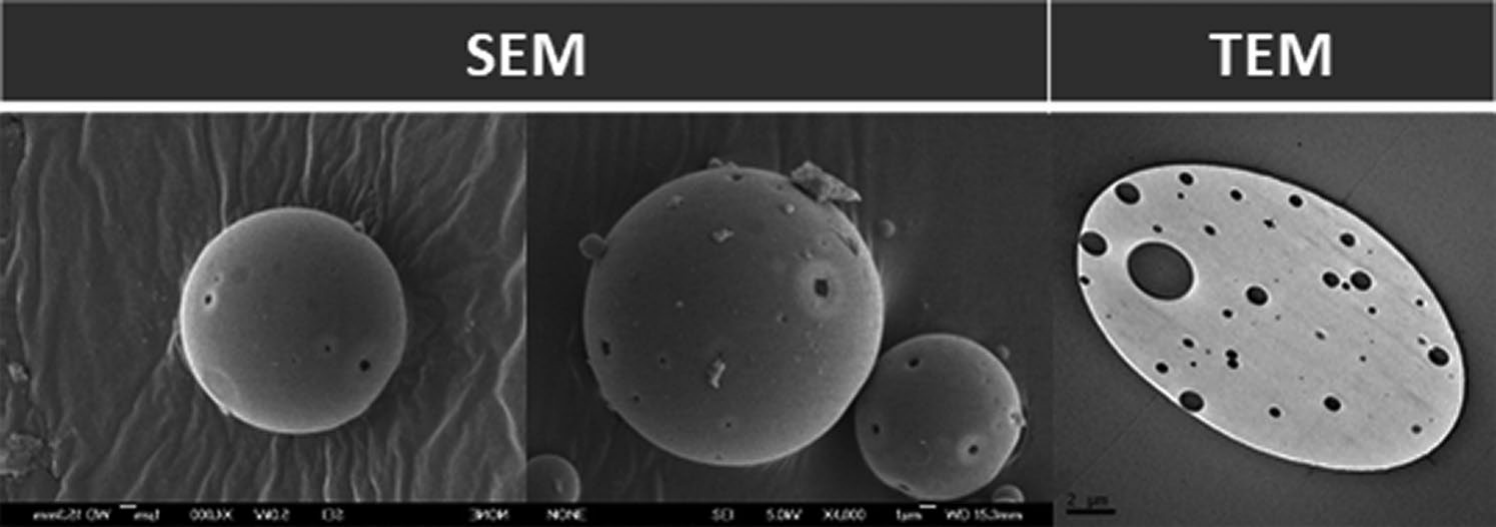
Dexamethasone-fibronectin co-loaded PLGA microspheres morphology. Scanning (left and center) and transmission (right) electron microscopy images.

SEM images evidenced the presence of spherical and regular-sized Ms with surficial porous and slightly rough surfaces. Moreover, the presence of internal porous was confirmed by TEM images, typically due to the double emulsion method employed for Ms elaboration (Figure 2).

### *In vitro* release studies

Both dexamethasone and fibronectin *in vitro* release profiles showed a multiphasic shape typically observed for PLGA microspheres.

**Figure 3.**
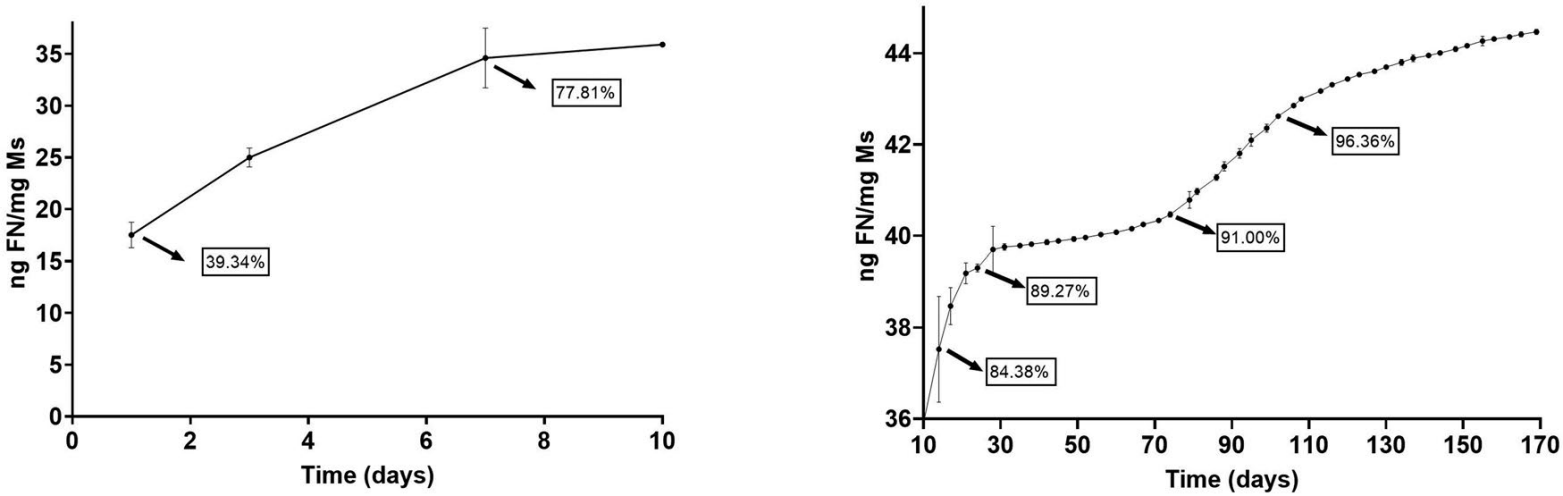
Cumulative *in vitro* release of fibronectin from dexamethasone-fibronectin co-loaded PLGA microspheres.

**Figure 4.**
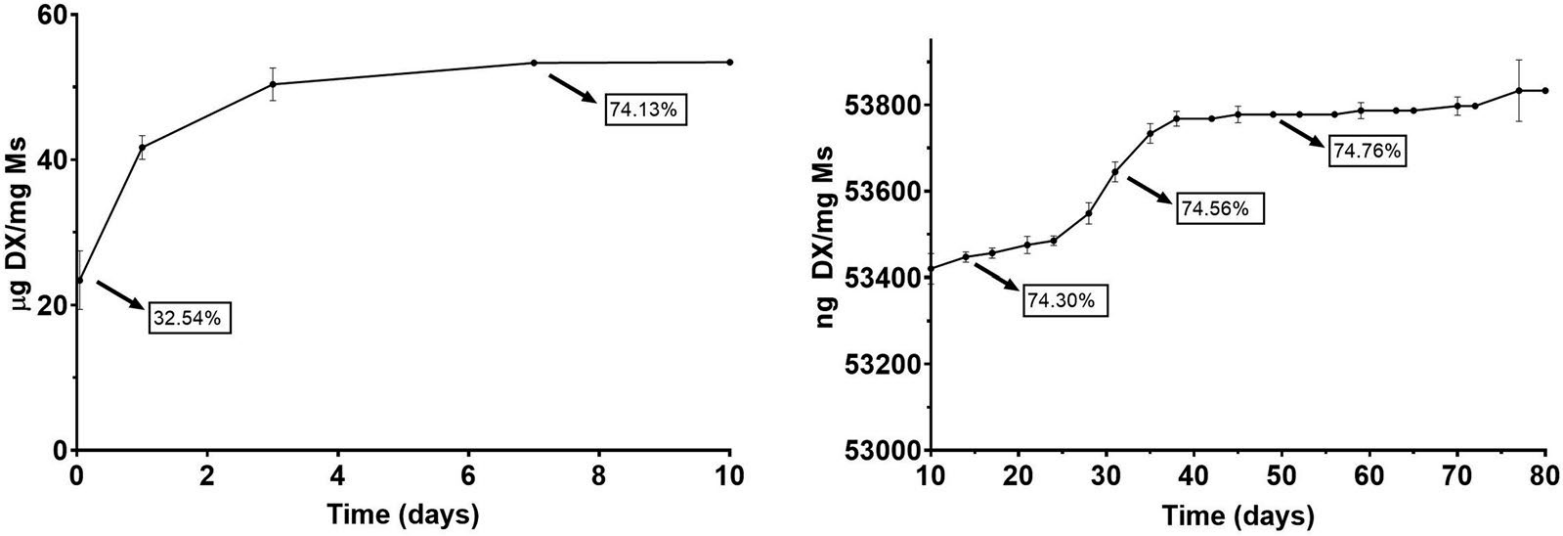
Cumulative *in vitro* release of dexamethasone from dexamethasone-fibronectin co-loaded PLGA microspheres.

Figures 3 and 4 show the cumulative amount of both fibronectin and dexamethasone per mg of Ms released versus time. In addition, to offer more complete information, values of the accumulated percentage of transfer are also provided throughout the graph.

Fibronectin underwent an initial rapid release of 35.90 ng/mg Ms in the first 10 days. After that, a sequence of rapid and slow-release profiles was detected: from day 10 to day 28, the profile shows a release of 0.211 ng FN/mg Ms/day, followed by a slower release of 0.017 ng FN/mg Ms/day from day 28 to 74. Next, from day 74 to 108, a second faster release of 0.074 ng FN/mg Ms/day was found and finally from day 108 to the end of the study the release rate was 0.024 ng FN/mg Ms/day.

Dexamethasone also showed a high initial release of 53.42 µg/mg Ms in the first 10 days and, as mentioned before, a subsequent slow but continuous release of different releases rates, characteristic of PLGA microspheres. From day 10 to 28, the *in vitro* release presented a slow phase with a rate of 7.22 ng DX/mg Ms/day, followed by a faster release of 22 ng DX/mg Ms/day from day 28 to 38 and the final release rate was slower, 1.54 ng DX/mg DX/day from day 38 to day 77. No dexamethasone was released from this time point.

### Ophthalmological signs and intraocular pressure

Forty of 43 animals used, did not show infection, intraocular inflammation, cataract formation or retinal detachment and the ocular surface was well preserved. The MsDexaFibro showed a tendency to localize at the superior iridocorneal angle, due to their lower density compared to aqueous humor and this disposition allowed a clear visual axis. One animal developed corneal leucoma and other an iridocorneal synechia that did not preclude proper testing and follow-up; however, another third rat developed a focus of vitreoretinitis so this animal was discarded and its results were not used.

**Figure 5.**
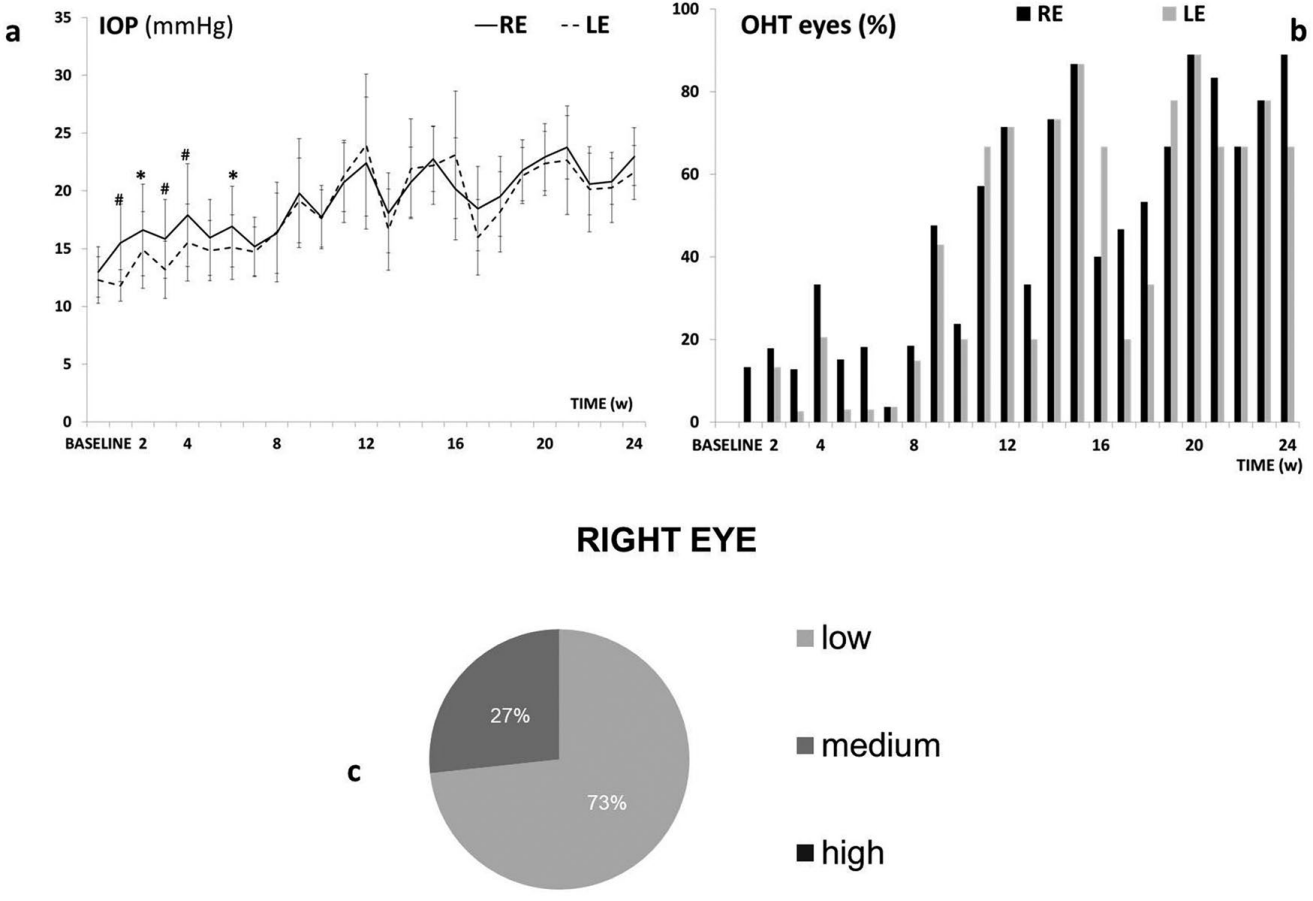
Intraocular pressure in the dexamethasone-fibronectin co-loaded microsphere (MsDexaFibro) model. a: Intraocular pressure curve over 6 months. b: Percentage of ocular hypertensive eyes (>20 mmHg). c: Percentage of corticosteroid response and tendency in right eyes over the study. d: Averaged percentage of corticosteroid response in right eyes. Low: <6 mmHg increase; medium: 6–15 mmHg increase; high: >15 mmHg increase. Abbreviations: IOP: intraocular pressure; RE: right eye; LE: left eye; w: week; OHT: ocular hypertension; %: percentage *: *p* < 0.05; #: *p* < 0.02 (Bonferroni Correction for multiple comparisons).

This model caused a progressive IOP increase and ocular hypertensive (OHT) eyes (>20 mmHg) over the study. The injected right eye (RE) showed higher measurements than the non-injected left eye (LE) up to 6 weeks (*p* < 0.05), but then both eyes similarly increased up to 24 weeks. Both eyes reached OHT at week 11 and fluctuations were observed over the study (Figure 5a). The highest percentage (88.9%) of OHT eyes was reached at 20 weeks (Figure 5b). Most rats experienced an IOP increase (in average) according to a medium corticosteroid response (between 6 and 15 mmHg), (Figure 5c) with constant lineal tendency on corticosteroid response in the injected eyes. Very few animals were high corticosteroid responders (>15 mmHg increase).

### *In vivo* neuroretinal examination

#### Electroretinography

**Figure 6.**
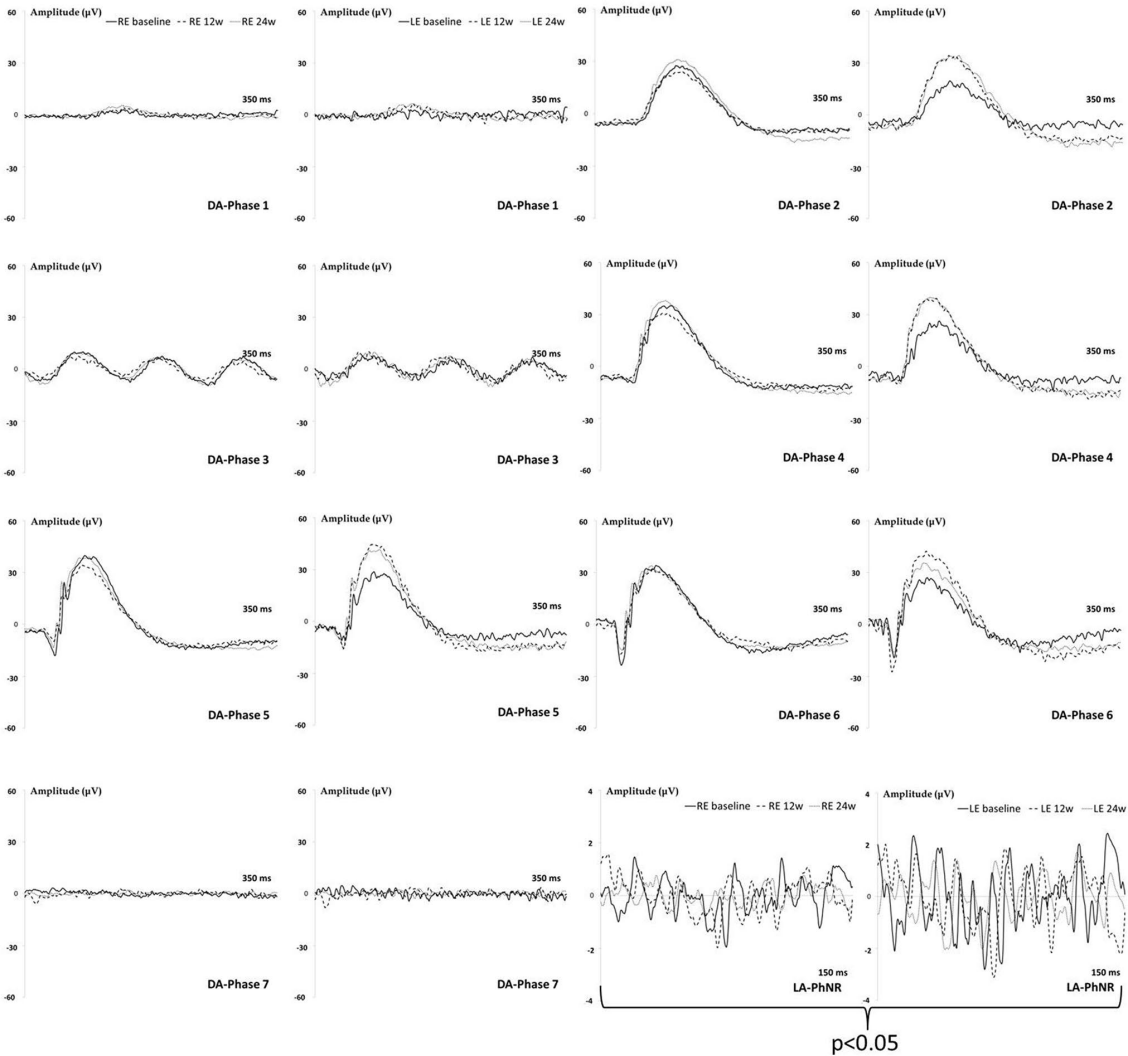
Neuroretina functionality, measured by dark- and light-adapted electroretinography (ERG), in the dexamethasone-fibronectin co-loaded microspheres (MsDexaFibro) model over 6 months of follow-up. MsDexafibro: microspheres loaded with dexamethasone and fibronectin; RE: right eye; LE: left eye; w: week; DA: dark-adapted; LA: light-adapted; μV: microvolts; ms: milliseconds.

The induced RE showed a tendency to longer latency signals in bipolar cells (b-wave) and smaller amplitudes in photoreceptors (a-wave) and bipolar (b-wave) cells compared to LE and over the study, but did not reach statistical differences with scotopic ERG conditions. However, the light-adapted PhNR protocol that specifically measures the retinal ganglion cells (RGC) activity detected smaller amplitudes (*p* < 0.05) in the injected RE, which it involves lower number of functional RGC (Figure 6).

#### Optical coherence tomography

**Figure 7.**
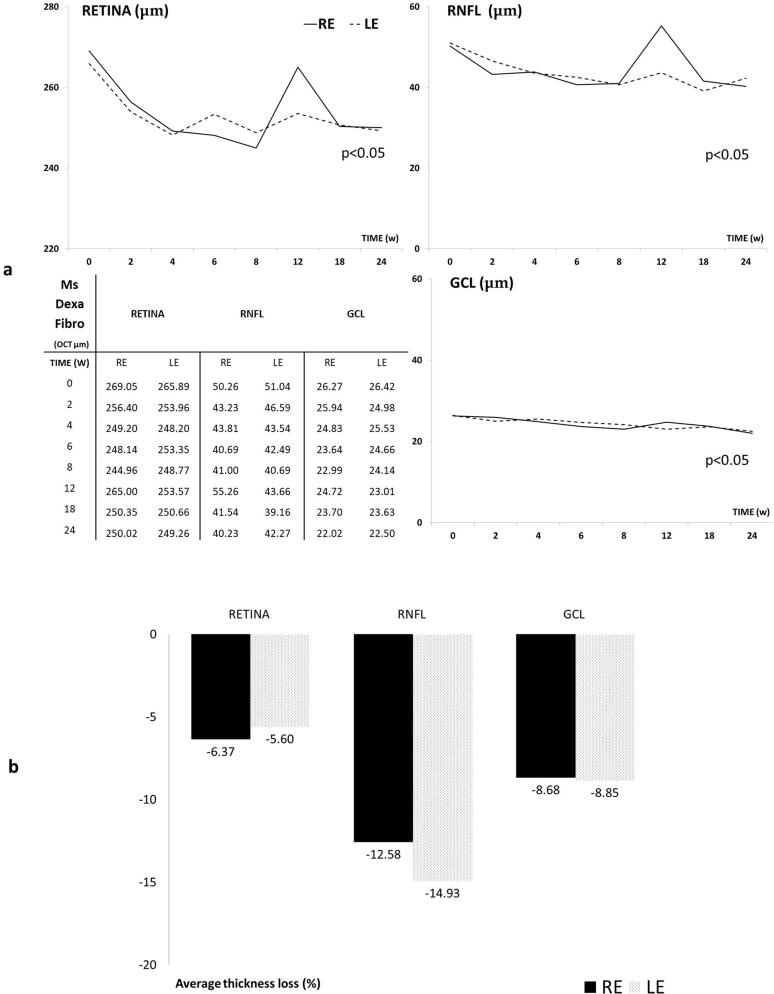
A Neuro-retinal thickness in microns by OCT throughout 6-month follow-up. B. Average thickness percentage loss by OCT in microspheres co-loaded with dexamethasone and fibronectin (MsDexaFibro) model up to 6-month follow-up. MsDexaFibro: microspheres co-loaded with dexamethasone and fibronectin; RE: right eye; LE: left eye; OCT: optical coherence tomography; RNFL: Retinal Nerve Fiber Layer; GCL: Ganglion Cell Layer complex; average thickness in microns (μm); w: week; %: percentage.

All the three R, RNFL and GCL protocols showed a progressive decrease in neuroretinal thickness over the study, with very few OCT sectors showed statistical significance (*p* < 0.05) between RE and LE, a tendency to lower thickness measurements was detected in the injected RE. Full retinal thickness (R) experienced the highest decrease followed by RNFL and GCL and it occurred in both eyes. And an increasing fluctuation was detected at week 12 especially in injected RE (Figure 7a). However, when the percentage of thickness loss over time was analyzed, the RNFL parameter showed the highest percentage loss at every time point explored and on average. It was followed by the GCL and finally the full retinal thickness. Injected RE showed a lower percentage loss of thickness in RNFL and GCL than the non-injected LE (Figure 7b).

The neuroretinal percentage loss by OCT sectors from RFNL, GCL and Retina was quantified and the loss tendency was analyzed. The inferior sector in RNFL showed the most intense and frequent loss. In GCL the inner sectors showed bigger percentage loss at any time explored in both eyes and the R experienced bigger loss in outer sectors with the STIN averaged loss trend (see Supplementary Figures S1–S3).

**Figure 8.**
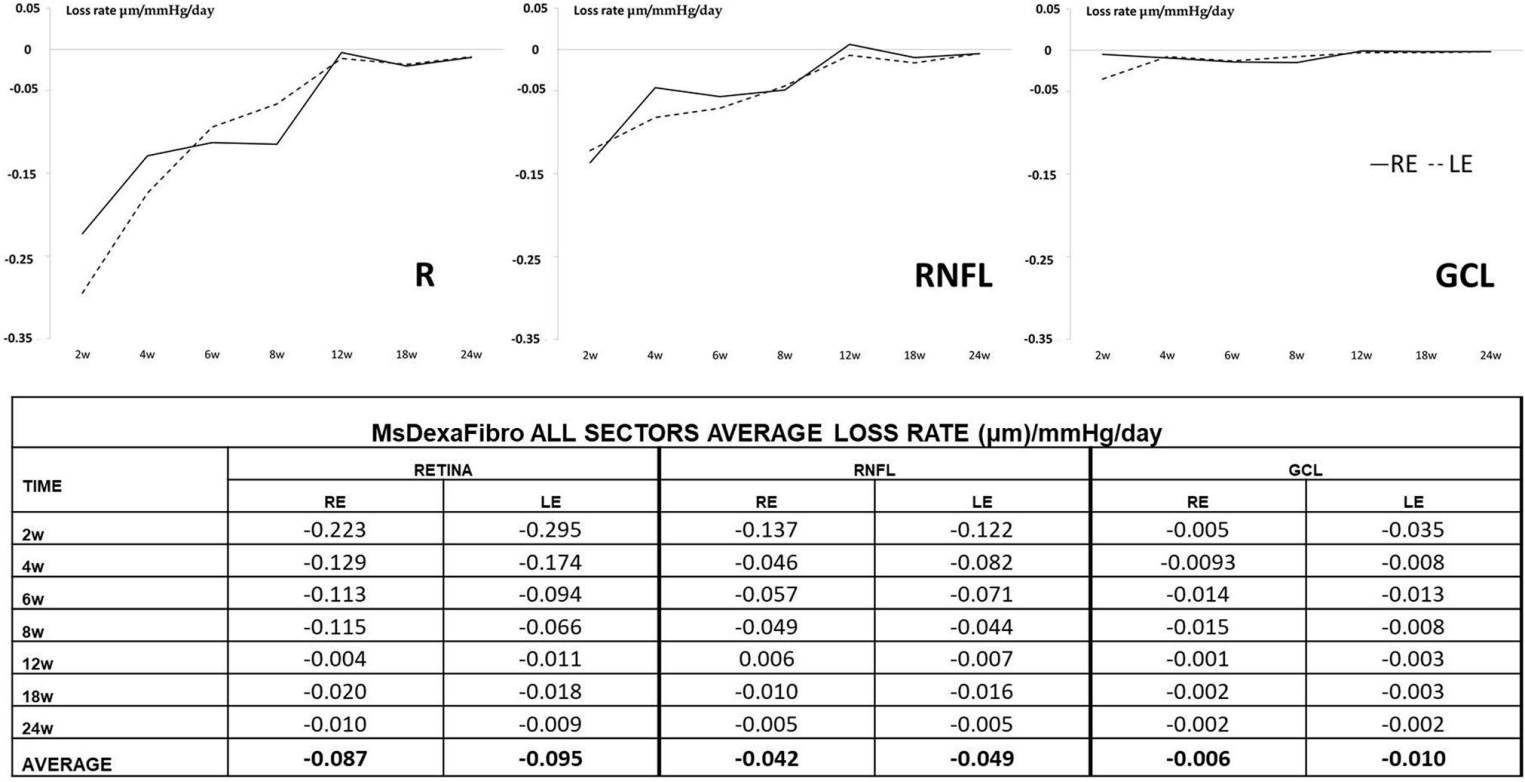
Neuro-retinal loss rate measured by optical coherence tomography (OCT) in the co-loaded with dexamethasone and fibronectin microspheres (MsDexaFibro) model. MsDexaFibro: microspheres co-loaded with dexamethasone and fibronectin; RE: right eye; LE: left eye; w: week; RNFL: Retinal Nerve Fiber Layer; GCL: Ganglion Cell Layer complex.

The average loss rate expressed in microns per mmHg and day extracted from all sectors was also quantified in both eyes and at all stages. Retina showed the highest loss rate followed by RNFL and finally GCL and the biggest loss rate occurred at early times. Moreover, both eyes lost similar quantity of microns in GCL thickness per every mmHg increased (Figure 8).

### Pathological findings

**Figure 9.**
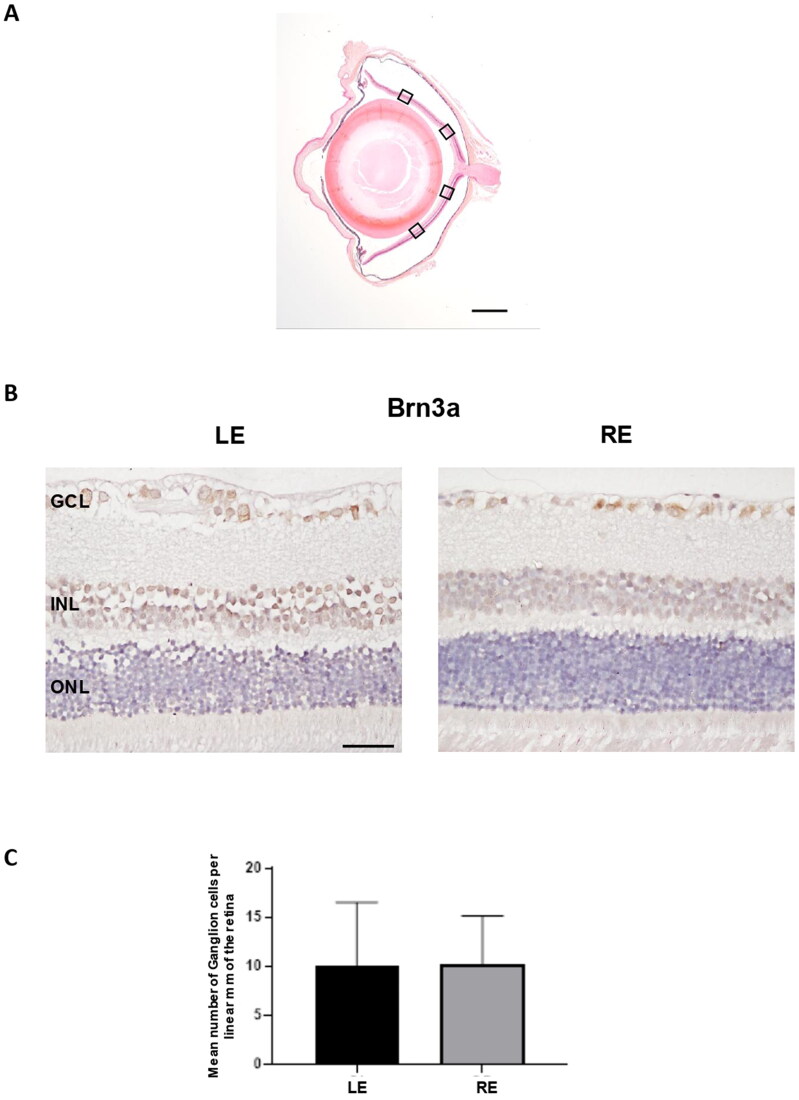
Ganglion cell analysis in glaucomatous eyes. A. Ganglion cells were counted in four areas (squares) of a radial section of the retina passing through the optic nerve. B. Two representative images of the retina marked with anti-Brn3a corresponding to a left non-intervened eye (LE) and a right eye (RE) injected with dexamethasone-fibronectin microspheres from the same animal. The mean number of ganglion cells per linear mm of retina was not different between non-intervened and injected eyes. GCL: Ganglion cell layer; INL: Inner nuclear layer; ONL: Outer nuclear layer. Scale bars: (A) 1 mm, (B) 30 µm.

**Figure 10.**
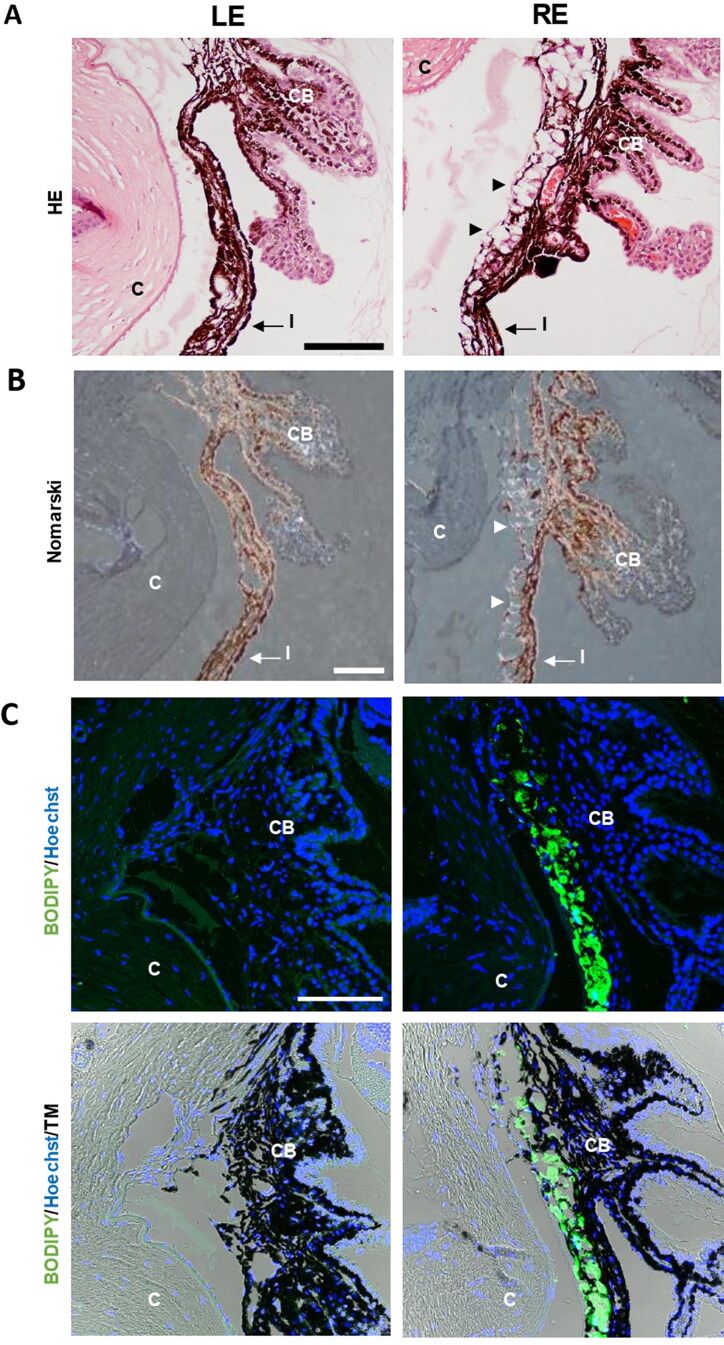
Detection of microspheres in injected eyes. A. Paraffin sections stained with Hematoxylin/Eosin exhibited the presence of the microspheres integrated into the iridocorneal angle (arrowheads), a finding never observed in the control ones. B. The characteristic light diffraction produced by microspheres using Nomarski microscopy (arrowheads) confirmed their presence in the iridocorneal angle of injected eyes. C. The Fluorescent Stain BODIPY (green) also showed the presence of microspheres hampering the iridocorneal angle and the trabecular meshwork. Nuclei were counterstained with Hoechst (blue). LE: left eye (non-intervened); RE: Right eye (dexamethasone-fibronectin microsphere injected). CB: Ciliary body; I: Iris; C: cornea; TM: Transmission mode. Scale bars: 100 µm.

The count of positive Brn3a cells along with four areas of the retina (Figure 9A), showed that the mean number of ganglion cells per linear mm of the retina was not significantly different in non-intervened left eyes in comparison to right eyes injected with dexamethasone-fibronectin microspheres (*p* = 0.92) (Figure 9B). However, fewer ganglion cells per linear mm of the retina were counted (10.12 ± 4.99) than in our previous model (12.16 ± 3.37) at 6 months (Rodrigo et al., 2021). In order to ascertain if the injected microspheres were located at the iridocorneal angle, they were analyzed using Hematoxylin/Eosin, Nomarski and fluorescent microscopy. The microspheres are transparent, and they are not stained by Hematoxylin or Eosin, but its presence can be still appreciated in the sections (Figure 10A). The characteristic light refraction produced by the microspheres was used to readily identify them in paraffin sections using Normarski microscopy (Figure 10B). The staining of microspheres with the fluorescent BODIPY, that we previously have demonstrated is specific for PLGA (Garcia-Herranz et al., 2021), further confirmed that injected microspheres are hampered the iridocorneal angle and the trabecula meshwork (Figure 10C).

**Figure 11.**
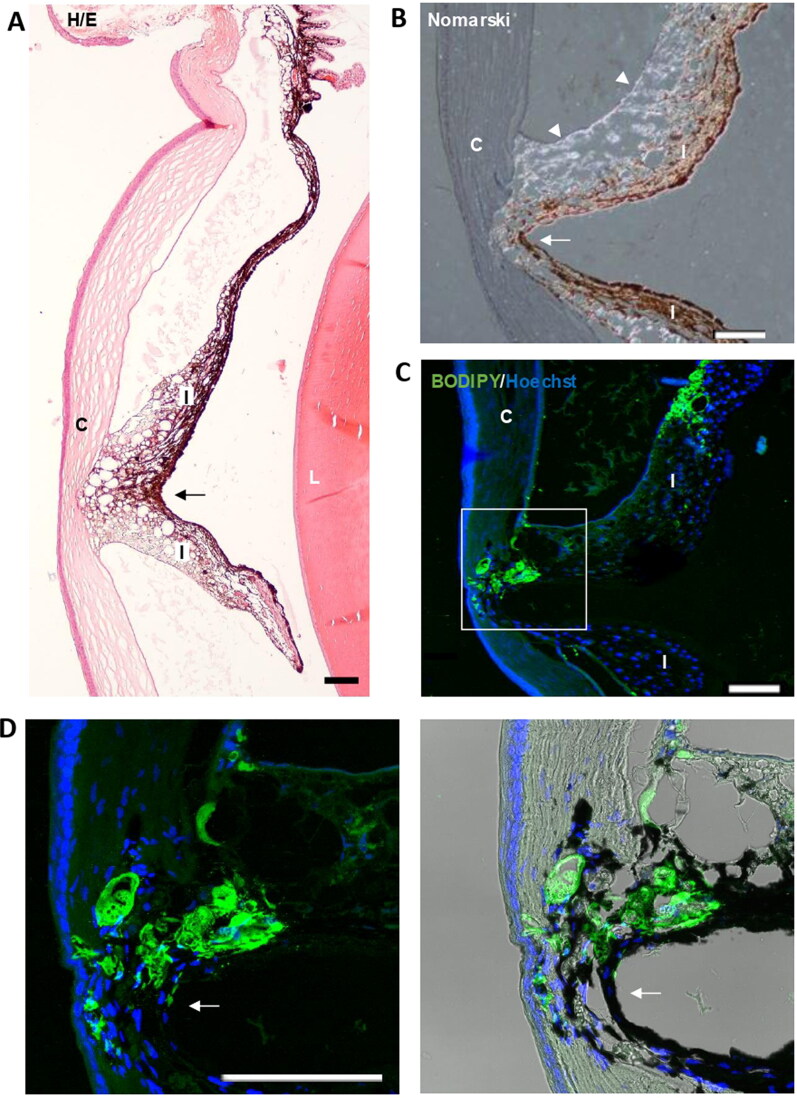
Iridocorneal synechias were observed in injected eyes (arrows). A. General view of a synechia between iris and the cornea. B. Using Nomarski microscopy, the diffracted light from the microspheres (arrowheads) was observed between the cornea and iris at the level of the focal contact between them. C. Staining with BODIPY (green) confirmed the presence of microspheres gluing the cornea and iris. Nuclei were counterstained with Hoechst (blue). C: cornea; I: Iris; L: lens; TM: Transmission mode. Scale bars: 100 µm.

Fibronectin plays a major role in the adhesion of many cell types, in fact, fibronectin is considered as the extracellular glue (Zollinger & Smith, 2017). Fibronectin has a RGD loop that is a promiscuous site for many cellular integrins, such as α5β1, α3β1, α8β1, and αvβ3 (Pankov & Yamada, 2002). This could explain how, unlike our other models where microspheres without fibronectin were injected (Garcia-Herranz et al., 2021; Rodrigo et al., 2021), focal adhesions were observed between the iris and the cornea (Figure 11). These synechiae are established through the fibronectin-containing microspheres and that would glue the iris and cornea (Figure 11). Obviously, these synechiae will modify the flow of the aqueous humor and therefore its reabsorption at the level of the trabecular meshwork.

## Discussion

The model presented in this work combines some of the strategies previously used in other rodent models of glaucoma, such as physical blockage of the trabecular meshwork, the administration of corticosteroids or the increased deposition of the protein fibronectin in the extracellular matrix of the trabecular meshwork (Roberts et al., 2020; Rodrigo et al., 2021). It was decided to co-release dexamethasone and fibronectin as the former stimulates extracellular matrix production and inhibits debris clearance (Dismuke et al., 2016; Zeng et al., 2020) and on the other hand, fibronectin deposition has been associated with ocular aging and glaucoma progression by playing an important role in trabecular meshwork occlusion in human glaucoma (Babizhayev & Brodskaya, 1989; Kasetti et al., 2017). In the model that we present, a combination of dexamethasone with fibronectin in PLGA microspheres is performed. This model progressively increased IOP over 24 weeks, reaching ocular hypertension at 11 weeks. There was a slight initial increase in IOP after induction, coinciding with the physical blockade of Ms at the iridocorneal angle and the first release of the encapsulated substances. Thereafter, IOP slowly increased and was maintained, coinciding with lower load release. It was probably due to extracellular matrix production by the constant presence of dexamethasone and the deposition of the protein fibronectin.

Microencapsulation and subsequent simultaneous release of more than one compound in modified release systems represent a technological challenge that has been developed in recent years as a very interesting strategy, for example, to be able to diminish the amount of polymer injected (Arranz-Romera et al., 2019; 2019). In order to encapsulate both compounds in the same formulation, it was decided to include each of them in the emulsion phase in which they were more soluble, so the protein was incorporated into the internal aqueous phase while dexamethasone was incorporated into the organic phase. In the recent past, there have been some efforts to encapsulate two or more substances in particles for different therapies. Although studies of multiloaded nanoparticles are more frequent (Jamil et al., 2019; Sokol et al., 2019; Madani et al., 2020), multiloaded microencapsulation is also being explored (Dutt & Khuller, 2001; Román et al., 2014; Shi et al., 2014; Baek et al., 2017). The main challenge of incorporating several substances is to accomplish a controlled release from those microsystems. In this sense, our research group has successfully achieved the microencapsulation and sustained release of more than one protein, the combination of proteins and low molecular weight compounds or even a tri-delivery of low molecular weight molecules using the single emulsion evaporation method (García-Caballero et al., 2018; Arranz-Romera et al., 2019; 2021). In the present work, we have achieved the release of both substances in a controlled and slow delivery for several months. Regarding double emulsions, works of co-encapsulation of two substances in the different phases of the emulsion are not very numerous. Qiao *et al* reported the co-microencapsulation of two antibiotics of different polarity by double emulsion, including each compound in the more soluble phase. They observed a slow release of both substances and suggested that the encapsulation of the hydrophilic compound may be enhanced with the addition of the hydrophobic one (Qiao et al., 2019). The same strategy has been successfully used in the present work; however, the potential benefit of the presence of the hydrophobic compound on the microencapsulation of the hydrophilic one has not been yet explored.

Obtaining models of neurodegenerative diseases of the retina is a challenge in the field of ophthalmology (Guidoboni et al., 2020). In the case of glaucoma, there are several animal models, among them, rodent animal models that are more convenient. Different mechanisms to create rat glaucoma models have been studied, as can be seen in Table 1, however, none of them achieves a slow and progressive degeneration of the retina as occurs when the disease develops in humans (Pang & Clark, 2020). In addition, neither of these rat models get an IOP increase for a long period of time. IOP-elevation induced glaucoma models (Dey et al., 2018) using non-biodegradable and biodegradable microspheres (Morgan & Tribble, 2015; Rodrigo et al., 2021; 2021) require periodic injections into the anterior chamber of the eye to maintain elevated pressures over prolonged study times. This fact increases the variability of results among different research groups, as well as the disadvantages derived from interventionism on the animal (Biswas & Wan, 2019). The present model, being single injection and technically simpler than other inducer models (Morrison et al., 2015; Dey et al., 2018), can be used by different research groups allowing for a more reliable comparison of treatments and therapies. It also improves animal welfare with reduced handling stress, reduces ocular complications such as infections or PLGA toxicity (Dossarps et al., 2015; Park, 2017; Zhao et al., 2017); as well as is more cost-efficient and generates less environmental impact (Haines et al., 2017).

**Table 1.** Pressure dependent glaucoma rat models.

Authors group and publication date	Mechanism of model creation	Sample size	Control eye	Average time for IOP to rise	Average time length of IOP increase	Average magnitude of IOP elevation from baseline	Feature of histology for retina at the study end
Urcola et al. (2006)	Intracameral injection microbead	*N* = 6	Contralateral eye and Control group	5 weeks	25 weeks	15 mmHg IOP increase	23% RGC death
Urcola et al. (2006)	Intracameral injection microbead with viscoelastic material	*N* = 4	Contralateral eye and Control group	6 weeks	24 weeks	20 mmHg IOP increase	27% RGC death
Samsel et al. (2011)	Intracameral injection of magnetic microbead	*N* = 61	Contralateral eye	After injection	12.8–27 days.	6 mmHg IOP increase	36% RGC cell loss
Moreno et al. (2005)	Intracameral administration of viscous agents	*N* = 45	Contralateral eye	Not specified	10 weeks	8–10 mmHg IOP increase	Significant RGC loss and damage in ON axons. Decrease in scotopic ERG activity
Morrison et al (1997) and Jia et al (2000)	Sclerosis of the outflow pathway by episcleral injection of hypertonic saline	*N* = 20	Contralateral eye	10 days	7–36 days	7–28 mmHg IOP increase	10–100% ON axon loss
Ueda et al (1998), WoldeMussie et al., 2001) Levkovith-Verbin et al. (2002)	Sclerosis of the outflow pathway by laser photocoagulation of outflow pathway	*N* = 10	Control group	1 week	5 weeks	6–24 mmHg IOP increase	50–70% ON axon loss
Shareef et al. (1995) Laquis et al. (1998)	Cautery of extraocular veins	*N* = 18	Contralateral eye	After injection	6 weeks–2.5 months	13–47 mmHg IOP increase	4% RGC loss per week
Sun et al. (2011)	Transient/intermittent IOP elevation by corneal limbus compression	*N* = 31	Contralateral eye	After compression	7 hours	25 mmHg IOP increase	52% RGC loss at 28 days after the insult
Morrison et al (2016)	Transient/intermittent IOP elevation by controlled elevation of IOP (CEI)	*N* = 122	Naïve group	After the insult	10 days	48 mmHg IOP increase	lesions in 83% of ON
Shepard et al (2010)	Transduction of the TM with glaucoma related genes by TGFβ2	*N* = 7	Contralateral eye	5 days	12 days	10–15 mmHg IOP increase	Not reported any potential RGC and ON axon loss.

Table extracted and adapted from Pang & Clark (2020). Abbreviations: ERG = electroretinogram. IOP = intraocular pressure. ON = optic nerve. RGC = retinal ganglion cells. TM = trabecular meshwork.

Our model showed that the most affected parameter by OCT was RNFL followed by inner sectors of GCL and finally full retina, which may reflect retrograde degeneration (Lawlor et al., 2018). Furthermore, when considering the GCL-RNFL component, the greatest standardized loss per mmHg was suffered by RNFL, again showing that the hypertensive noxa exerts early and greater damage to axonal structure (Howell et al., 2007; Guo et al., 2010). At earlier times, the Inferior and Nasal sectors of RNFL seem to be the most vulnerable to damage, and at later times they become ISNT (INFERIOR > SUPERIOR > NASAL > TEMPORAL glaucoma rule) due to the possible influence of dynamic pathogenic changes. An example is the important fluctuation at 12 weeks of the study observed in this model of glaucoma as well as observed in our previous one and in healthy animals linked to neuroplasticity and glia involvement (Rodrigo et al., 2020; 2021; 2021). In the GCL, the sectors most affected on average were the inner sectors TSN (TEMPORAL > SUPERIOR > NASAL), which coincide with those with the highest density of retinal ganglion cells (RGC) (Salinas-Navarro et al., 2009; Guo et al., 2010). Glaucoma mainly affects the RGCs, and their decreased functionality has been detected in photopic conditions with the PhNR or pattern ERG tests, and scotopic conditions with pSTR (Porciatti, 2015; Wilsey et al., 2017). Furthermore, outer retinal cells changes have also been detected in scotopic conditions with reversible high IOP (approx. 35 mmHg) (Choh et al., 2016), and in patients with late glaucoma (Graham et al., 1995). In the present model, where the damage occurred gradually, RGC dysfunction with a statistically significant decrease in PhNR was detected early (12 weeks); and coinciding with mild hypertensive levels (20–22 mmHg) a limitation of outer retinal function was also found at early-intermediate stages.

A bilateral neurodegeneration after induction of unilateral OHT has been already observed in several animal models (Sapienza et al., 2016; de Hoz et al., 2018; Rodrigo et al., 2021; 2021). This can be explained because the glial activation, characteristic of glaucomatous eyes, spreads through the visual pathway, so the degenerative factors could, in turn, activate the glia of the contralateral eye (Howell et al., 2007), producing retrograde damage and also creating an imbalance in pressure at the optic nerve head (Jóhannesson et al., 2018). In addition, in other works a gradual increase in IOP in the non-induced contralateral eye has been also detected (Roubeix et al., 2015; Sapienza et al., 2016; Rodrigo et al., 2021; 2021), as in our case. In fact, the use of the contralateral eye as a control is not recommended in glaucoma animal models.

The present study shows a rat model of glaucoma with ocular hypertension that is easy to induce; with a high likelihood of being reproducible, since only one induction and nature-modifying intervention is performed; biologically plausible with minimal side effects, as the injected material is biocompatible as it degrades to CO_2_ and H_2_O in the Krebs cycle (Martins et al., 2018) and its trabecular meshwork modifying compounds (dexamethasone and fibronectin) are present features of steroid-induced glaucoma and primary open-angle glaucoma (Rozsíval et al., 1981; Babizhayev & Brodskaya, 1989; Kasimov & Aghaeva, 2017). This model try to mimics then the pathophysiology of human glaucoma, with predictable IOP increase and progressive neuroretinal loss, by avoiding rapid increases in OHT and severe neuroretinal loss (Agarwal & Agarwal, 2017). All this fulfills the characteristics of an ideal model and allows for long-term studies (Biswas & Wan, 2019).

The animal model presented in this work is a clear example of how pharmaceutical technology can help other disciplines to achieve optimized results by joining forces. While the usual employ of controlled drug release systems is focused on the treatment of pathologies, we present in this paper another important utility of sustained-release systems, in this case not to correct damage to the body but to cause it. The use of modified-release systems to obtain animal models is very poorly developed yet but, as observed in this work, it presents enormous potential. Regarding this idea and consistent with a lower total dose of dexamethasone than that used in our previous model, mostly mild levels of corticoresponse were found. But, a similar % of rats (approx. 90%) developed OHT, and even fewer ganglion cells per linear mm of retina (10.12 ± 4.99 vs 12.16 ± 3.37) were counted at 6 months (Rodrigo et al., 2021). This supports the important role of fibronectin in the development of OHT.

Neuroprotective therapies aims to protect neurons from secondary degeneration, already described in the introduction, which is the true trigger for the amplification of neuronal degeneration in chronic retinopathies (Cheung et al., 2008). Therefore, in order to correctly evaluate neuroprotective therapies, it is necessary to start from experimental animals with healthy retinas and that these retinas slowly degenerate, as occurs in human retinal chronic pathologies (Morrison et al., 2011). The rat model that is presented in this work would allow this type of study since, by achieving a very progressive ocular hypertension and sustained over time, the degeneration of the retina is also so. In addition, one of the main problems of the *in vivo* evaluation of intraocular drug delivery systems is that it is very difficult to demonstrate the potential of the system when applied on retinas as degenerated as those that appear in most animal models of glaucoma (Nadal-Nicolás et al., 2016; Rodrigo et al., 2021). In this sense, our animal model, by offering a prolonged degeneration of the retina over at least 6 months, would be a very useful candidate to evaluate this type of modified release system. In the present model, only an initial injection is needed to produce the sustained damage and, furthermore, this injection takes place in the anterior chamber of the eye, without any other structure of the eye being affected. Thus, the intravitreal space remains intact to test any modified release system for long periods.

Concerning the limitations of this work and future studies, (I) Due to the lability of fibronectin, encapsulation could not be quantified and therefore the actual amount of fibronectin injected is unknown. However, *in vitro* release was in the order of nanograms, much lower than *in vivo* results of fibronectin quantifications in the aqueous humor of glaucoma patients (Kim et al., 1992), which we did not perform in our study in order not to alter the model by adding other puncture and maintaining eye homeostasis as much as possible; (II) We have not analyzed the differences by sex, even when its involvement is linked to immune-mediated pathologies (Desai & Brinton, 2019; Rodrigo et al., 2021) and glaucoma; (III) It was neither evaluated the potential immune role of the protein fibronectin nor the anti-inflammatory effect of dexamethasone on it (Hernandez et al., 2020; Mzyk et al., 2022) and that it would be interesting to study in future works; (IV) Finally, glaucoma is an aged-link pathology, and the rats were in their childhood at the start of the study, so results could deviate from translational human interpretation.

In conclusion, a new chronic glaucoma animal model was created by a single injection of PLGA co-loaded MsDexaFibro very similar to open-angle glaucoma. This chronic rat model would have an important impact in ophthalmology, so it allows long periods of study of this pathology, in pharmacology because it could be used to evaluate the neuroprotective activity of active compounds and also in pharmaceutical technology, so it could allow the correct evaluation of the efficacy of sustained intraocular drug delivery systems.

## Supplementary Material

Supplemental Material
