# Supplementary material for: Mimicking chronic glaucoma over 6 months with a single intracameral injection of dexamethasone/fibronectin-loaded PLGA microspheres
Source: Drug Deliv. 2022 Jul 29;29(1):2357–74. doi: 10.1080/10717544.2022.2096712 (PMC9341346; doi:10.1080/10717544.2022.2096712)

**Supplementary figure 1.** **Retina nerve fiber layer (RNFL) percentage loss by optical coherence tomography (OCT) sectors and loss trend in MsDexafibro model over 6 months**. RE: right eye; LE: left eye; w: week; TV: total volume; S: superior; I: inferior; N: nasal; T: temporal.**
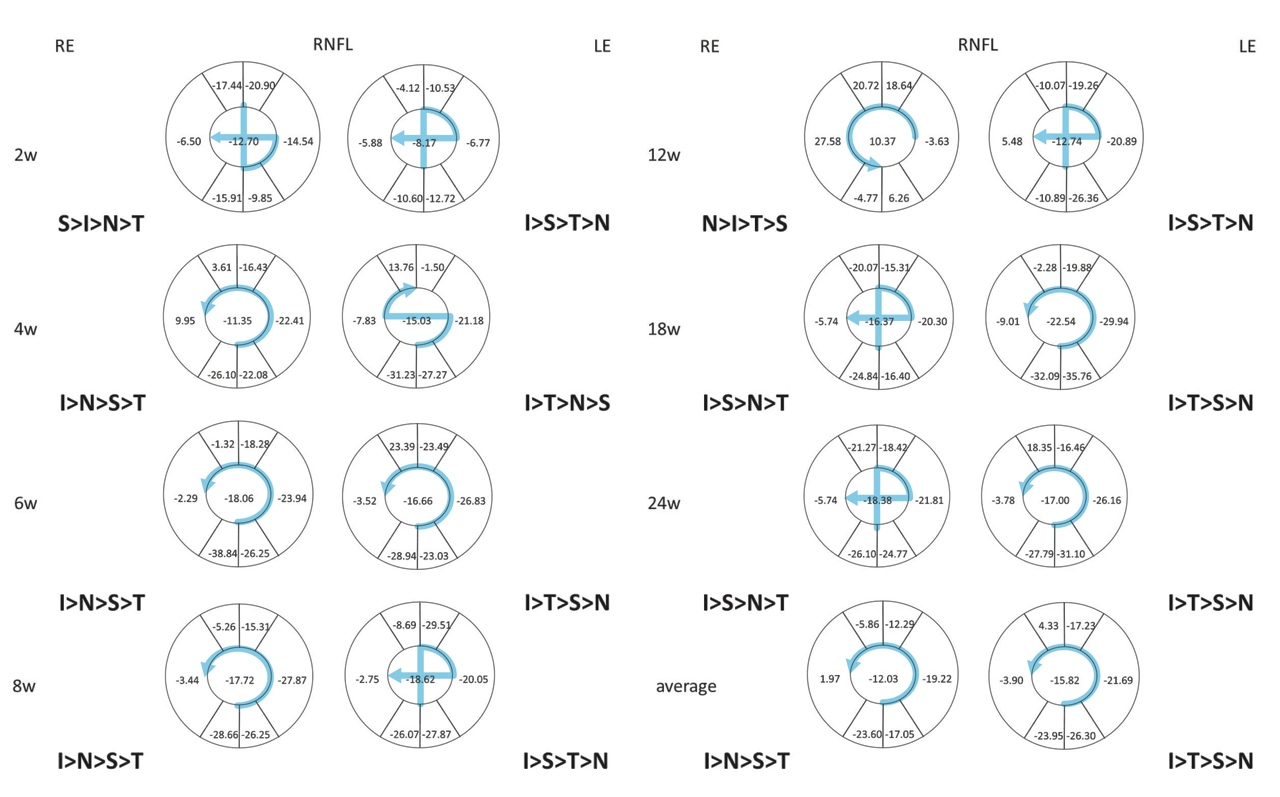
**

**Supplementary figure 2.** **Ganglion cell layer (GCL) percentage loss by optical coherence tomography (OCT) sectors and loss trend in MsDexafibro model over 6 months.** RE: right eye; LE: left eye; w: week; TV: total volume; S: superior; I: inferior; N: nasal; T: temporal.


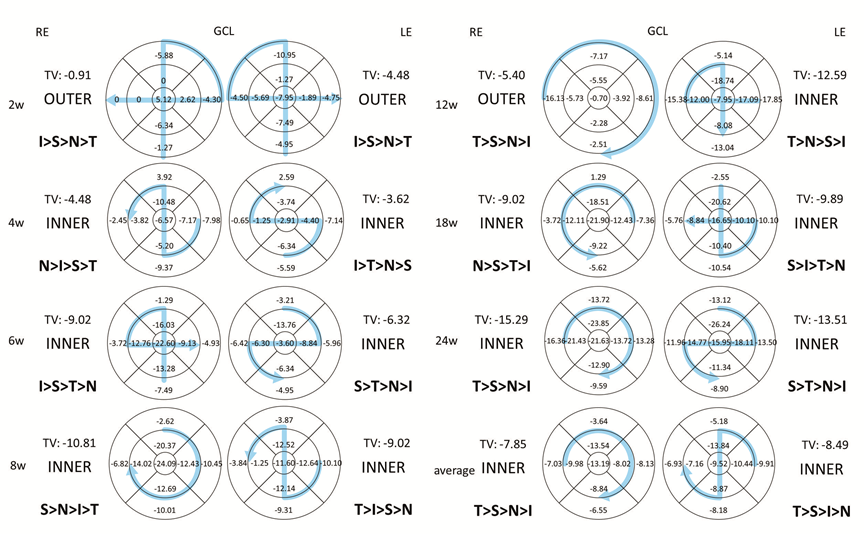


**Supplementary figure 3.** **Retinal** **percentage loss by optical coherence tomography (OCT) sectors and loss trend in MsDexafibro model over 6 month.** RE: right eye; LE: left eye; w: week; TV: total volume; S: superior; I: inferior; N: nasal; T: temporal.


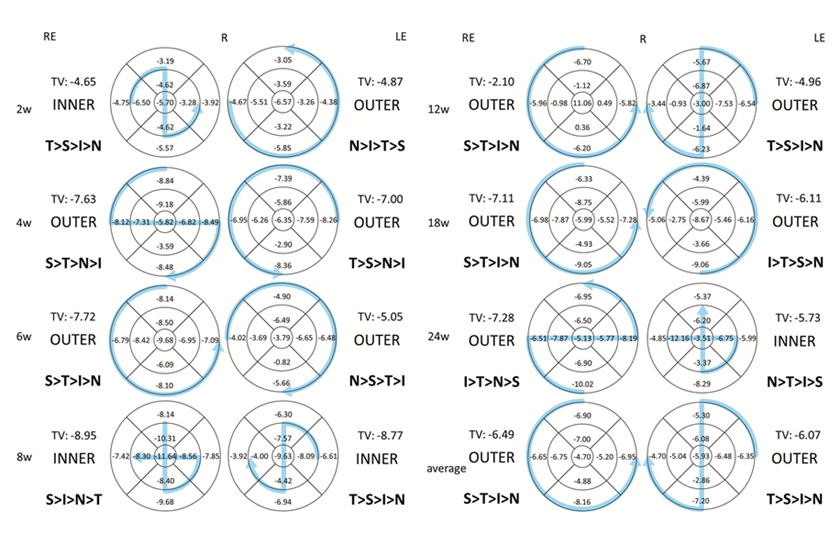

Supplement: Supplemental Material [file IDRD_A_2096712_SM5424.docx]
